# Current Landscape of Preclinical Models for Pediatric Gliomas: Clinical Implications and Future Directions

**DOI:** 10.3390/cancers17132221

**Published:** 2025-07-02

**Authors:** Syed M. Faisal, Monika Yadav, Garrett R. Gibson, Adora T. Klinestiver, Ryan M. Sorenson, Evan Cantor, Maria Ghishan, John R. Prensner, Andrea T. Franson, Kevin F. Ginn, Carl Koschmann, Viveka Nand Yadav

**Affiliations:** 1 Department of Pediatrics, Children’s Mercy Research Institute (CMRI), Kansas City, MO 64108, USA; 2 Division of Hematology and Oncology, Connecticut Children’s Medical Center, Hartford, CT 06106, USA; 3 Department of Pediatrics, Division of Pediatric Hematology/Oncology, University of Michigan Medical School, Ann Arbor, MI 48109, USA; 4 Department of Pediatrics, University of Missouri-Kansas City School of Medicine, Kansas City, MO 64108, USA; 5 Department of Cancer Biology, University of Kansas Cancer Center, Kansas City, KS 66160, USA

**Keywords:** pHGGs, diffuse midline gliomas, immunocompetent mouse model of DMGs, in utero electroporation, GEMMs

## Abstract

Pediatric high-grade gliomas (pHGGs), particularly diffuse midline gliomas (DMGs), are among the most lethal brain tumors due to poor survival and resistance to therapies. DMGs possess a distinct genetic profile, primarily driven by hallmark mutations such as H3K27M, ACVR1, and PDGFRA mutations/amplifications and TP53 inactivation, all of which contribute to tumor biology and therapeutic resistance. Developing physiologically relevant preclinical models that replicate both tumor biology and the tumor microenvironment (TME) is critical for advancing effective treatments. This review highlights recent progress in in vitro, ex vivo, and in vivo models, including patient-derived brain organoids, genetically engineered mouse models (GEMMs), and region-specific midline organoids incorporating SHH, BMP, and FGF2/8/19 signaling to model pontine gliomas. Key genetic alterations can now be introduced using lipofectamine-mediated transfection, PiggyBac plasmid systems, and CRISPR-Cas9, allowing the precise study of tumor initiation, progression, and therapy resistance. These models enable the investigation of TME interactions, including immune responses, neuronal infiltration, and therapeutic vulnerabilities. Future advancements involve developing immune-competent organoids, integrating vascularized networks, and applying multi-omics platforms like single-cell RNA sequencing and spatial transcriptomics to dissect tumor heterogeneity and lineage-specific vulnerabilities. These innovative approaches aim to enhance drug screening, identify new therapeutic targets, and accelerate personalized treatments for pediatric gliomas.

## Simple Summary

Pediatric high-grade gliomas (pHGGs) remain one of the most challenging cancers to treat effectively. Developing accurate and reliable preclinical models is crucial for understanding tumor biology and testing potential therapies. This review article provides a comprehensive overview of the current strategies used in preclinical models for pediatric glioma, highlighting their strengths and limitations. It also outlines a roadmap for current and future model development to overcome existing challenges and leverage novel opportunities.

## 1. Introduction

Pediatric brain tumors represent the most common solid tumors in children and are a major cause of cancer-related deaths [1,2]. Although significant progress has been made in cancer treatments, survival rates for patients with central nervous system (CNS) tumors remain dismal [3]. Pediatric high-grade gliomas (pHGGs) now surpass leukemia as the most prevalent cancer type [3,4,5,6]. The Central Brain Tumor Registry of the United States reports an annual age-adjusted incidence rate of 5.83 per 100,000 among children aged 0–14, with a mortality rate of 0.71 per 100,000 as of 2020 [1,4,7].

Advances in imaging, molecular diagnostics, and treatment have revealed the complexity and diversity of pediatric brain tumors, underscoring the limitations of histological classification alone [8]. Tumors with similar histological features often exhibit distinct molecular profiles, resulting in varied prognoses and treatment responses [9]. In 2016, the World Health Organization (WHO) redefined tumor classification to incorporate molecular diagnostic criteria, a transition furthered within the 2021 WHO Classification of Pediatric Tumors, which integrates morphological, immunohistochemical, and molecular characteristics [10,11,12].

Although most pediatric brain tumors occur sporadically, certain genetic syndromes predispose children to their development. These genetic predispositions include neurofibromatosis types 1 and 2 (*NF1*, *NF2*), Li–Fraumeni syndrome (*TP53*), and Gorlin syndrome (*PTCH1*), among others [13,14]. Genomic studies have revealed distinct tumor subtypes based on their molecular landscapes. For example, medulloblastomas are frequently associated with mutations in *CTNNB1*, *PTCH1*, *MYC*, and *MYCN*, while the H3K27M mutation characterizes diffuse midline gliomas (DMGs) [14,15,16,17,18,19,20]. Recent advancements in genomic and proteomic studies have significantly deepened our understanding of pediatric brain tumors [8,15,16,21,22,23]. For instance, genomic profiling of diffuse intrinsic pontine gliomas (DIPGs) has revealed three distinct molecular subgroups, H3K27M, silent, and *MYCN*, each with unique genetic drivers and therapeutic vulnerabilities [15]. Notably, mutations in the activin receptor gene *ACVR1* were identified in approximately 20% of DIPGs, leading to constitutive activation of downstream signaling pathways [15]. In medulloblastomas, single-cell transcriptomic analyses have uncovered subgroup-specific cellular heterogeneity, with *WNT*, *SHH*, Group 3, and Group 4 tumors exhibiting distinct developmental trajectories and cellular origins [22]. Additionally, highly aggressive *MYC*-subgroup medulloblastomas, characterized by c-MYC overexpression, have been modeled in mice, providing a robust platform to study their unique pathobiology and develop targeted therapies [23].

Recent advances in molecular profiling have significantly deepened our understanding of therapeutic resistance mechanisms in pediatric high-grade gliomas (pHGGs), particularly in diffuse midline gliomas (DMGs). Single-cell RNA sequencing has revealed that H3K27M-mutant DMGs retain neurodevelopmental programs, where malignant cells remain trapped in progenitor-like states that resist differentiation and promote therapeutic failure [17]. Spatial transcriptomic studies further demonstrate distinct tumor cell states and microenvironmental organization across patient ages and tumor locations, implicating developmental context and adaptive stress responses as central drivers of heterogeneity and resistance [24]. Both immune evasion and metabolic rewiring play major roles in resistance, often linked to these developmental programs and tumor-intrinsic stress responses [25]. Alterations in oxidative metabolism, epigenetic dysregulation, and immunosuppressive microenvironments contribute to the resilience of these tumors. Novel immune-competent genetically engineered mouse models (GEMMs), immune-competent organoid platforms, and single-cell atlases have emerged as essential tools for functionally interrogating tumor–immune interactions within the complex tumor microenvironment (TME). Despite these advances, multi-omics studies specifically clarifying tumor–immune interactions in DMGs remain underrepresented. Initial transcriptomic profiling has shown that tumor-secreted cytokines and chemokines recruit immunosuppressive myeloid cells while excluding cytotoxic T cells, fostering immune evasion [17,24]. Early spatial proteomic studies have begun mapping these immune niches, revealing localized microglial activation that supports tumor growth [26]. Meanwhile, clinical advances demonstrate the efficacy of GD2- and B7-H3-targeted CAR T-cell therapies in overcoming immune escape mechanisms, offering promising avenues to disrupt immune privilege in these tumors [27,28]. Ongoing clinical trials are actively evaluating agents like panobinostat, veliparib, and oncolytic virotherapy, highlighting the translational momentum to overcome these multi-layered resistance mechanisms [29,30,31]. Collectively, these findings underscore that pHGG and DMG resistance is orchestrated by developmental misprogramming, metabolic plasticity, and immune evasion, emphasizing the need for integrative, multi-omics, immunologic, and precision-based therapeutic strategies.

Proteomic studies have also shed light on the tumorigenic mechanisms of the H3K27M mutation in DMGs. This mutation disrupts PRC2 complex activity, leading to epigenetic dysregulation, reduced chromatin accessibility, the widespread loss of H3K27me3 with genome depression and increased noncoding genome transcription, and the transcriptional repression of neurodevelopmental genes [16,32]. These findings highlight the role of epigenetic reprogramming and enhancer dysfunction in driving tumorigenesis, paving the way for epigenetic-targeted therapies. Together, these insights into molecular subgroups, epigenetic mechanisms, and cellular heterogeneity are transforming our understanding of pediatric brain tumors and guiding the development of novel, targeted treatment strategies.

Despite these advancements, outcomes for pHGGs remain dismal. Median overall survival (OS) for patients with H3K27M-mutant DMG is approximately 9 to 12 months from the time of diagnosis [8,15,17,21,33]. Common neurologic findings in patients with DMG include headaches, vision changes, difficulties with balance, memory issues, and, less commonly, seizures. Chemotherapy regimens expose patients to many toxic side effects such as a suppressed immune system, fatigue, and gastrointestinal distress. Pediatric low-grade gliomas (pLGGs) and recurrent ependymomas have a progression-free survival of <50% [5,8,11,12]. Immunotherapy offers new hope for pediatric brain tumors, but its success has been limited by the immunosuppressive tumor microenvironment (TME) and the restrictive blood–brain barrier (BBB) [27,28,34,35]. Combination therapies targeting multiple aspects of tumor biology and immune activation may overcome these challenges, as evidenced by ongoing preclinical research [6,36,37,38,39,40,41,42,43]. Research since 2008 has linked pHGGs to neural and oligodendrocyte precursor cells (NPCs and OPCs), identifying genetic drivers such as histone mutations [44]. The complex heterogeneity, with diverse genetic, epigenetic, and cellular profiles, contributes to the therapeutic resistance and poor outcomes of pHGGs.

The WHO’s 2021 glioma classification reflects a paradigm shift, emphasizing molecular features in combination with traditional histology [10]. This tumor classification system separates pediatric and adult gliomas into distinct entities and incorporates TME, heterogeneity, and glioma stem cell (GSC) dynamics into therapeutic strategies. Despite advances, most high-grade gliomas remain resistant to standard therapies, necessitating innovative preclinical models.

Pediatric brain tumors, particularly high-grade gliomas (pHGGs), diffuse midline gliomas (DMGs), and medulloblastomas, exhibit profound molecular heterogeneity, which significantly impacts disease behavior, treatment response, and prognosis. This heterogeneity presents a critical challenge for preclinical model development: traditional models often fail to recapitulate the molecular complexity of human tumors, limiting translational relevance [45,46].

To maintain clinical applicability, it is essential to select or engineer in vivo and in vitro models that accurately reflect the molecular landscape of the specific tumor subtype under investigation [17,47]. For example, genetically engineered mouse models (GEMMs) or in utero electroporation (IUE)-based models incorporating H3K27M and ACVR1 mutations provide faithful recapitulation of diffuse midline gliomas, including their epigenetic and invasive characteristics [48,49,50]. Similarly, patient-derived xenografts (PDXs) and patient-derived cell lines maintain key genetic and epigenetic alterations present in original tumors and enable the study of inter-patient variability in drug response [51].

Thus, integrating knowledge of molecular heterogeneity into model selection is critical for generating translationally relevant preclinical data, guiding the development of personalized therapies for these devastating pediatric brain tumors.

Preclinical models are critical for understanding tumor biology and evaluating drug pharmacokinetics, pharmacodynamics, and tumor-specific responses [6,52,53,54,55]. However, the blood–brain tumor barrier (BTB) poses significant obstacles to drug delivery [34]. Advanced tissue-engineering platforms, such as patient-derived xenografts (PDXs), genetically engineered mouse models (GEMMs), human cerebral organoids, and microfluidic systems, provide accurate tools to study tumor dynamics, heterogeneity, and therapeutic responses [56,57,58,59,60,61,62]. These platforms enable the development of therapies tailored to the unique molecular landscapes of gliomas, paving the way for more personalized treatments.

Recent advances in pediatric neuro-oncology include avapritinib (PDGFRA/KIT inhibitor), which elicited tumor shrinkage in three of seven patients with PDGFRA-altered high-grade glioma and is now in a Phase 1/2 trial [63]; GD2- and B7-H3-targeted CAR T therapy delivered intracerebroventricularly, which has earned FDA Breakthrough and RMAT designations after demonstrating safety, repeated-dose feasibility, and survival benefit in DIPG, with a Phase 2 trial planned [27,64]; ^124I-omburtamab, which, via convection-enhanced delivery, achieved localized, well-tolerated delivery in DIPG [65]; TRX-E-009-1, which, in combination with SAHA and radiation, restored H3K27me3, induced apoptosis, and significantly extended survival in DIPG preclinical models [66]; repurposed therapeutics—mebendazole, bumetanide, and carmofur—which have shown blood–brain barrier penetration and cytotoxicity in pediatric glioma models [67]; 186Re nanoliposomal radiotherapy via convection-enhanced delivery, which is in a Phase 1 study for DIPG, ependymoma, and HGG (Plus Therapeutics ReSPECT-PBC); and a UF Health-led mRNA-LNP immunotherapy, which is in early-phase testing for recurrent pediatric HGG. Prunin, a plant-derived flavonoid, shows emerging potential as an anticancer agent with the ability to enhance conventional therapies and reduce resistance. It would be interesting to investigate its efficacy in pediatric high-grade glioma models [68].

Emerging modeling techniques, including 3D bioprinting and genome engineering, further enhance the ability to replicate tumor biology [8,55,69,70,71,72]. While no model is perfect, these approaches offer critical insights into tumor heterogeneity and treatment efficacy [8,70,71,72,73,74], bridging the gap between experimental findings and clinical translation.

## 2. Molecular Classification of Malignant Pediatric Brain Tumors

Molecular classification has revolutionized the understanding of pediatric brain tumors (Table 1). By categorizing tumors based on genetic and molecular characteristics, researchers can identify specific subtypes that may respond differently to treatments [10,11,12,15,75,76,77,78]. This section highlights the molecular subtypes of pediatric brain tumors, including medulloblastomas, gliomas, and ependymomas, and discusses their clinical implications for prognosis and treatment [79].

Pediatric brain tumors differ significantly from their adult counterparts as they arise from distinct precursor cell types [21,80,81]. These biological differences contribute to varied responses to treatment, and treatments found to provide survival benefits in adults have not necessarily led to similar improvements in children [82,83]. For instance, therapies initially optimized for adults, such as radiation therapy, can disrupt the developing brain in children, particularly those under the age of five [17,21]. Although radiation therapy is a mainstay of treatment for many pediatric brain tumors, there is significant attention paid to the age of the child at the initiation of radiation and the high likelihood of long-term sequelae should the patient survive past early childhood.

Historically, CNS tumors were classified based on histology, anatomical location, and morphological similarities to specific cell types [7,84,85,86]. However, the incorporation of genomic technologies into clinical practice has significantly advanced this classification [10]. Molecular profiling, powered by techniques like next-generation sequencing (NGS) and single-cell RNA sequencing, has provided a detailed understanding of the genetic and epigenetic landscapes of brain tumors. These approaches have uncovered critical differences between pediatric and adult brain tumors, even when their histology appears similar [12,17,36,75,87,88,89,90]. Refinements of classification systems, such as the 2016 WHO CNS Tumor Classification, now integrate molecular features to improve alignment between treatment strategies and patient prognoses. Over time, pediatric-specific classifications have emerged, incorporating key molecular hallmarks such as histone mutations, DNA methylation patterns, and kinase signaling pathway alterations [8,15,17,43,75,76,87,89,90,91,92,93,94].

### 2.1. pHGGs

These tumors frequently harbor histone H3 mutations, including K27M and G34R/V, which define distinct subtypes [95]. Tumors harboring the H3G34R/V mutation are located primarily in the cerebral hemispheres, particularly within the cortex and subcortical white matter, and are most commonly classified as diffuse hemispheric gliomas (DHGs). These tumors predominantly arise in the supratentorial region, particularly in the frontal, parietal, or temporal lobes. They are more frequently observed in adolescents and young adults and are characterized by frequent TP53 mutations and ATRX alterations contributing to genomic instability. Notably, unlike H3K27M-mutant tumors, H3G34R/V-mutant gliomas do not involve midline brain structures [16,17,46,48,70,87,95,96,97]. Other pHGG subtypes include those that are *ACVR1*-mutant, histone wild-type, such as pleomorphic xanthoastrocytoma (PXA)-like gliomas enriched with BRAF mutations, and NF1-associated gliomas with receptor tyrosine kinase fusions (Table 1) [42,98,99,100,101]. Despite these genetic insights, pHGGs remain challenging to treat, with limited therapeutic options and poor survival outcomes.

### 2.2. DIPG/DMG, H3K27M-Mutant

**Figure 1:**
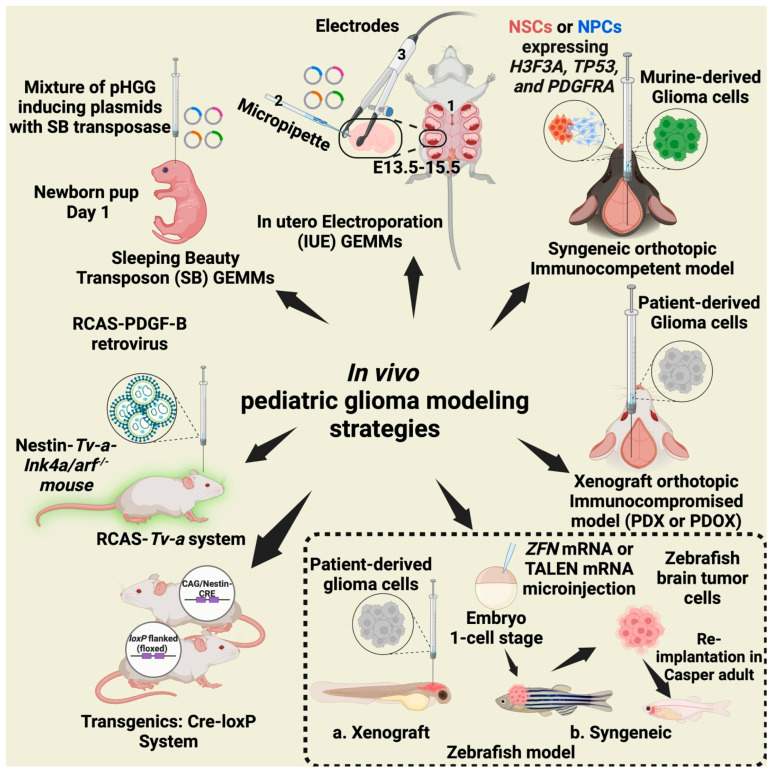
In vivo pediatric brain tumor modeling strategies. Multiple in vivo approaches have been developed to model pediatric high-grade gliomas (pHGGs), including diffuse midline gliomas (DMGs), to better understand tumor biology and therapeutic responses. Among these, in utero electroporation (IUE)-based GEMMs have emerged as a powerful system, enabling precise delivery of glioma-driving genetic alterations into neural progenitors during embryonic development, resulting in tumors that closely mimic the molecular and histopathological features of human disease. Additional models include Sleeping Beauty (SB) transposon-based approaches and RCAS-Tv-a and Cre-loxP transgenic systems, as well as orthotopic transplantation of murine-derived or patient-derived glioma cells into immunocompetent or immunocompromised mouse brains. Zebrafish models utilize xenograft (a) and syngeneic (b) strategies, including ZFN (zinc finger nuclease) or TALEN (transcription activator-like effector nuclease) mRNA injection into 1-cell embryos or the re-implantation of zebrafish brain tumor cells into adult Casper fish for tumor progression studies (created with BioRender.com).

**Figure 2:**
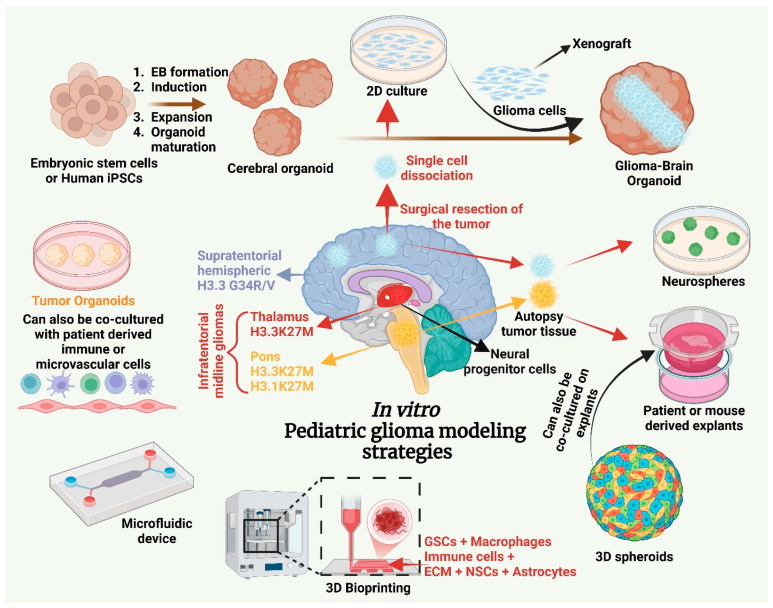
In vitro pediatric glioma modeling strategies. Advanced in vitro platforms, including cerebral organoids, tumor-derived organoids, neurospheres, and 3D spheroids, are used to model pediatric high-grade gliomas (pHGGs) and their tumor microenvironment (TME). Glioma–brain organoids, microfluidic devices, and 3D bioprinting further enable dynamic modeling of tumor–immune–brain interactions and therapeutic responses. These models also facilitate the study of region-specific gliomas with distinct genetic mutations, such as H3K27M or H3G34R (created with BioRender.com).

DIPGs are diagnosed radiographically and, upon tumor tissue characterization, are characterized as DMGs. H3K27M-mutant DMG represents one of the most aggressive forms of pediatric cancer. These tumors typically arise in midline structures such as the pons, thalamus, and spinal cord and are defined by specific histone H3K27M mutations in *H3F3A* (H3.3), *HIST2H3B/D* (H3.2), or *HIST1H3B/C* (H3.1) where lysine is substituted with methionine at position 27 (K27M) (Table 1; Figure 1 and Figure 2) [40,46,87,89,90,102]. Notably, approximately 80% of DMG cases exhibit a recurrent somatic mutation in H3F3A or HIST1H3B, resulting in K27M substitution and driving widespread epigenetic dysregulation [103]. The H3K27M mutation in DMGs drives epigenetic dysregulation by inhibiting the enzymatic activity of the EZH2 subunit of the PRC2 complex, resulting in a global reduction in H3K27me3 and uncontrolled oncogenesis and tumor progression. In addition to the H3K27M mutation, these tumors frequently exhibit alterations such as TP53 mutations and PDGFRA mutations/amplifications, further driving their aggressive nature [37,50,90,104,105]. DMGs pose a significant clinical challenge due to their highly aggressive biological behavior and marked resistance to conventional therapies. Over 200 clinical trials of standard cancer therapies failed to show efficacy in DMG [106]. Significant surgical resection is generally not feasible due to the tumor’s critical location within midline brain structures, such as the brainstem and thalamus, and its diffuse, infiltrative growth pattern. Radiation therapy remains the primary palliative treatment, potentially providing temporary symptomatic relief. Unfortunately, survival rates remain dismal, as tumor progression occurs in nearly 100% of cases [107]. Advances in molecular profiling and preclinical models offer hope for the development of more effective, personalized therapies to improve outcomes for patients with this devastating disease. Given that a large percentage of cases harbor the H3K27M mutation, therapies targeting this specific mutation represent a promising avenue for treatment [108].

### 2.3. Infant-Type Hemispheric Gliomas

These rare tumors exhibit favorable clinical outcomes despite their high-grade histological appearance [10,77,109]. They are frequently associated with MAPK pathway alterations resulting from fusion genes involving *ALK*, *ROS1*, *NTRK*, and *MET* [100,109,110]. These tumors often have better prognosis and may be responsive to receptor tyrosine kinase inhibitors. These often-targetable genetic alterations provide significant opportunities for targeted therapeutic approaches.

### 2.4. pLGGs

pLGGs are the most common pediatric brain tumors and are often associated with favorable survival outcomes, especially following complete surgical resection. pLGGs are driven predominantly by alterations in the MAPK signaling pathway, including BRAF-KIAA1549 fusions, BRAF V600E mutations, and FGFR1 alterations [42,76,78,97,111]. Pilocytic astrocytomas, the most common pLGG subtype, frequently harbor BRAF fusion, while diffuse pLGGs may be associated with FGFR1 mutations. Many patients with pLGGs, including pilocytic astrocytoma, will undergo surgery (either biopsy, partial resection, or total resection) followed by some form of therapy. The standard of care is chemotherapy, generally a low-toxicity regimen with vincristine/carboplatin, though targeted therapies are increasingly being used both at relapse and more recently as upfront therapy [78]. Targeted therapies inhibiting the MAPK pathway offer promising alternatives to traditional treatments and are being investigated for use as upfront therapies [41,76]. While MAPK pathway inhibitors have shown efficacy in pLGGs, their success in pHGGs has been limited. The more aggressive biology of pHGGs, including additional oncogenic mutations, widespread epigenetic reprogramming, and a highly immunosuppressive microenvironment, creates resistance mechanisms that hinder MAPK inhibitor effectiveness. Moreover, blood–brain barrier permeability and adaptive pathway activation further reduce therapeutic impact in pHGGs, necessitating combinatorial approaches for improved efficacy.

### 2.5. Oligodendrogliomas

Pediatric oligodendrogliomas are rare and distinct from their adult counterparts. While adult oligodendrogliomas are defined by IDH mutations and 1p/19q co-deletions, pediatric oligodendrogliomas often lack these molecular hallmarks [112]. Instead, they commonly harbor FGFR1 mutations or single oncogenic alterations [72,113,114,115].

### 2.6. Ependymomas

Ependymomas are rare gliomas that develop from the ependymal cells lining the brain’s ventricular system [8,11]. In children, these tumors predominantly occur in the posterior fossa and exhibit distinct molecular profiles, such as PFA and PFB subtypes in posterior fossa ependymomas and RELA-fusion positivity in supratentorial ependymomas (Table 1) [86,88,116,117]. PFAs and PFBs are characterized by DNA hypomethylation and CpG island hypermethylation, which silence genes involved in chromatin modification [88,92,118,119]. Treatment involves surgical resection and radiotherapy, but these tumors have a high recurrence risk and poor survival outcomes.

### 2.7. Integration of Molecular and Histological Insights

Advances in molecular profiling have identified ten distinct subgroups of pediatric gliomas, enabling improved classification, patient stratification, and personalized therapeutic strategies. Genetic and epigenetic mutations, such as histone alterations, are often correlated with specific tumor biology, providing critical insights for targeted therapies. For example, gliomas harboring BRAF alterations, including fusions and V600E mutations, exhibit distinct biological behaviors. BRAF fusions are observed predominantly in pediatric-type gliomas and are associated with MAPK pathway activation. In contrast, the BRAF V600E mutation, while targetable with BRAF/MEK inhibitors, does not universally indicate a less aggressive phenotype, particularly in pHGGs. Clinical studies have shown that while pLGGs with BRAF alterations tend to respond favorably to targeted therapy, pHGGs with BRAF V600E mutations may still exhibit aggressive behavior despite targeted inhibition (Table 1) [120,121,122]. These findings emphasize the importance of incorporating molecular diagnostics into clinical practice to optimize outcomes for pediatric patients with brain tumors. Emerging technologies, including spatial transcriptomics and organoid-based modeling, offer promising tools to unravel the biological complexity of pediatric gliomas further (Figure 2). Combining molecular insights with advanced preclinical models holds significant potential for identifying novel therapeutic approaches tailored to the specific biology of these tumors [123].

## 3. In Vivo Models of pHGG

Reliable animal models are essential for studying pediatric brain tumors. These models should achieve high tumor incidence, replicate the histopathological and molecular features of human tumors, and reflect treatment responses observed in clinical settings (Figure 1). A variety of preclinical models have been developed to explore tumor biology, study microenvironmental interactions, and evaluate potential therapies [4,46,48,51,55,70,71,73,124,125,126,127,128,129,130,131,132,133,134,135,136]. Rodent models, particularly mice and rats, are widely used due to their versatility [46,71,130,137,138,139]. However, zebrafish models are gaining attention as an alternative because of their advantages, such as rapid tumor formation and ease of genetic manipulation, making them ideal for large-scale studies [140,141,142,143,144].

### 3.1. Carcinogen-Induced Animal Models

Carcinogen-induced models are critical tools for brain tumor research, especially in rats, which exhibit a higher efficiency of tumor induction compared with mice. These models utilize chemical carcinogens, such as N-nitrosourea and its derivatives, which can induce diverse tumor types, including gliomas, astrocytomas, oligodendrogliomas, and ependymal tumors [4,52,145]. Developing embryos are especially sensitive to carcinogens, and the compounds are often administered via transplacental injection during gestation [146]. For example, injecting ethylnitrosourea at gestational day 20 into pregnant rats reliably induces brain tumors in all offspring [147,148,149]. Chemical carcinogens can also be administered postnatally through oral, intravenous, or local routes. However, repeated administration is often necessary to enhance tumor induction, particularly in older animals due to the reduced sensitivity of mature brain tissues to carcinogens. Several cell lines, including CNS1, C6, T9, 9L, BT4C, F98, and RG2, have been derived from these models and are extensively employed for preclinical studies focusing on brain tumor biology and therapeutic testing [150].

### 3.2. Oncogenic Virus-Induced Models

Brain tumors can be induced experimentally using specific oncogenic viruses, including RNA viruses such as Rous sarcoma virus-1 (RSV-1) and DNA viruses like human adenoviruses [145,151,152]. For instance, injecting RSV-1 into the brain of neonatal rodents can result in malignancies, with different characteristics depending on the site of injection [153]. Similarly, human adenovirus 12 (Ad12) has been shown to cause CNS tumors in various species, including hamsters, mice, and rats [153,154]. Tumor incidence rates differ among these species, with higher frequencies observed in rats (91.0%) compared with mice (30.2%) and hamsters (37.2%) [153]. The brain regions most commonly affected include the olfactory bulb, lateral ventricular horns, tapetum, and fourth ventricle, as well as the spinal cord, particularly the dorsal root and cauda equina in hamsters [152,154]. Additionally, subependymal regions and leptomeningeal tissues frequently develop micro-tumors. From a histological perspective, Ad12-induced tumors are largely undifferentiated, resembling remnants of perinatal subependymal cells [154]. These tumors display diverse cellular arrangements, such as spongioblastic cells forming fascicular or palisading structures, with occasional perivascular pseudorosettes and neuroblastic rosettes [155,156]. More complex rosette formations, such as ependymoblastomatous and medulloepitheliomatous types, are less commonly observed [157]. Research indicates that tumors induced by Ad12 originate from embryonal neuroectodermal cells, which exhibit restricted differentiation potential. These tumors give rise to a range of phenotypes, including medulloblastoma, neuroblastoma, primitive spongioblastoma, ependymoblastoma, and, in rare cases, medulloepithelioma [158,159]. While carcinogen- and virus-induced glioma models have contributed to our understanding of gliomagenesis, their direct relevance to pediatric high-grade gliomas (pHGGs) is limited. These models often produce heterogeneous tumors with variable morphology and molecular profiles, and typically do not recapitulate hallmark pHGG mutations such as H3K27M or H3G34R/V. In contrast, genetically engineered mouse models (GEMMs), in utero electroporation (IUE)-based models, and patient-derived orthotopic xenografts (PDOXs) are specifically designed to incorporate clinically relevant driver mutations and faithfully reproduce the molecular, epigenetic, and invasive features of pHGG. Thus, while virus- and carcinogen-induced models remain valuable for studying general mechanisms of gliomagenesis or tumor–host interactions, GEMMs and PDOXs are superior platforms for modeling pHGG-specific biology and evaluating the efficacy of new treatment strategies.

### 3.3. Xenograft Animal Models

Xenograft models involve transplanting human-derived tumor cells or tissues into immunocompromised animals, serving as a valuable system for investigating tumor biology and evaluating therapeutic strategies [4,33,51,125,126,134,160,161,162]. Furthermore, emerging approaches using neural stem cells (NSCs) or neural progenitor cells (NPCs) engineered to overexpress oncogenes have shown promise in generating accurate preclinical models of DMGs [48,163,164]. These models enable the investigation of tumor progression, therapeutic targets, and epigenetic dysregulation associated with pHGGs. Xenograft models can be categorized into cell line-derived xenografts (CDXs), PDXs, and patient-derived orthotopic xenografts (PDOXs).

Although CDX models have been widely used for gliomas, they exhibit significant limitations when applied to pHGGs [138,165,166]. These cell lines are expanded in vitro and subsequently implanted into immunodeficient mice, such as NOD/SCID or NSG strains, through intracerebral injection. The key advantages of CDX models include high reproducibility, rapid tumor formation, and the ability to interrogate specific pathways and drug responses linked to pHGGs. These models are widely used to study genetic drivers, signaling pathways, and tumor vulnerabilities (Table 2). However, CDX models present notable limitations. They often fail to replicate the heterogeneity and cellular complexity observed in pHGGs, which are characterized by significant inter- and intra-tumoral variation [167]. Additionally, prolonged in vitro culture of cell lines in monolayer systems can lead to aberrant characteristics, including abnormal collagen expression, altered integrin patterns, and dysregulated immune markers, resulting in genomic and transcriptomic divergence from the original pediatric tumors [4,52,168].

PDX and PDOX models address limitations of traditional xenografts by better retaining the molecular and genetic features of pHGGs, including driver mutations like H3K27M and H3G34R/V, making them highly relevant for translational research [4,169]. While subcutaneous implantation of PDX models offers practical benefits, it fails to replicate the critical TME observed in pHGGs. In contrast, orthotopic PDOX models provide a more physiologically relevant system by mimicking the tumor’s native microenvironment [57]. These models enable the study of key features of pHGG biology, including tumor progression, therapy resistance, and immune interactions, and treatment responses. By implanting tumor cells or spheroids directly into the original neuroanatomical site (e.g., pons or thalamus) as the original tumor [42,161,170], PDOX models exhibit high fidelity in preserving histopathological and genetic characteristics. Importantly, these models are not subjected to in vitro artifacts, maintaining the genomic and epigenomic integrity and phenotypic complexity of the parental tumors, including hallmark mutations like H3K27M, and the global loss of H3K27me3 [42,51,171]. Despite variability in engraftment rates and tumor latency, PDOX models serve as a valuable platform for evaluating novel therapies, radiosensitizers, and combination treatments in a setting that reflects clinical reality [42,161,170].

Various methods are employed to prepare tumor cells for injection, including the dissociation of neurospheres or the direct isolation of cells from surgical samples. Tumor cells can also be enriched for brain tumor-initiating cells (BTICs) using methods such as CD133+ cell sorting [42,162,172,173]. While subcutaneous propagation preserves tumor traits and reduces the establishment time for models, intracranial implantation remains the preferred approach for studying brain tumors due to its ability to better replicate the tumor’s native environment. Rigorous validation of PDX models is essential, involving thorough histological and molecular analyses to ensure faithful representation of the original tumors (Table 2). To enhance the standardization and reproducibility of PDX models, initiatives such as the Pediatric Preclinical Testing Consortium and the Childhood Solid Tumor Network have been launched [174,175]. These consortia focus on the systematic collection and validation of PDX models, adhering to the PDX Minimal Information standard (PDX-MI), which defines key clinical data and experimental procedures required to maintain quality and consistency [176]. Despite challenges in accessing patient data and ensuring privacy, these collaborative efforts have underscored the significance of validated PDX models in advancing pediatric brain tumor research.

## 4. Immune-Competent Pediatric Brain Tumor Models

In pediatric glioma studies, various in vivo models are utilized, including GEMMs and viral delivery models. GEMMs have the potential to replicate tumor initiation in vivo by supporting BBB integrity, with viral delivery models acting as a less-complex substitute for GEMMs. Immune-competent pediatric brain tumor models are crucial for accurately studying tumor–immune interactions and evaluating immunotherapies. These models retain an intact immune system, allowing the assessment of CAR-T, checkpoint inhibitors, and other immune-modulating therapies. They should mimic the TME, maintain genetic and epigenetic fidelity (e.g., H3K27M, ACVR1 mutations), and support proper tumor localization for clinical relevance. Syngeneic models, such as IUE and Sleeping Beauty (SB) GEMM models, enable immune response studies while preserving tumor characteristics, making them essential for translational research in pediatric brain tumors. These models are further essential for understanding tumor mechanisms and discovering new drugs (Table 2 and Figure 1).

### 4.1. GEMMs

GEMMs are indispensable for investigating the early events of tumor initiation, particularly in systems with an intact immune microenvironment and BBB. These models allow researchers to study tumor progression under physiological conditions that more closely mimic the human disease [8,50,55,70,71,72,73,177]. In pediatric glioma research, GEMMs frequently incorporate mutations that disrupt key signaling pathways, including Ras, EGFR, Akt, Rb, PTEN, NF1, and PDGF signaling [4,8,72,89,178]. Early GEMM platforms focused on mutations in tumor suppressor genes such as Nf1 and Trp53. By crossbreeding mice with varying genetic backgrounds, these models recapitulate the progression from low-grade astrocytoma to high-grade gliomas [178]. For instance, introducing CNS-specific heterozygosity of *PTEN* into *NF1*/*p53* knockout mice accelerates tumor growth, culminating in aggressive high-grade astrocytomas [4,179,180,181]. To improve targeting specificity, advanced systems like conditional or inducible knockout GEMMs were developed. One commonly utilized approach is the Cre-loxP system, enabling researchers to study tumor progression at different stages and in specific tissues, mimicking the progression of gliomas in humans more accurately [182]. For example, astrocyte-specific expression of oncogenic V12Ha-Ras using the GFAP promoter has successfully modeled astrocytomas resembling the human disease [183]. More recently, inducible spontaneous tumor models of pHGG have been utilized to study H3K27M mutations and their interplay with other genetic aberrations, providing critical insights into pHGG pathogenesis [24,184,185,186]. Fortin et al. (2020) have recently demonstrated that spontaneous tumor models of ACVR1 elucidate the role of the mutant ACVR1^G328V^ in arresting oligodendroglial lineage differentiation. When combined with *HIST1H3B*^K27M^ and *PIK3CA*^H1047R^, this mutation subsequently drives the gliomagenesis of pHGGs [101].

### 4.2. Viral Delivery Models

Viral delivery systems present a less-complex and -labor-intensive alternative to traditional GEMMs. A notable example is the RCAS/t-va system, which facilitates the targeted delivery of oncogenic genes to somatic cells. This method enables gene expression in a small, defined subset of cells, effectively modeling the early stages of tumor development [133]. Several pediatric brain tumor models have been generated using this system [45,187]. In a landmark study by Becher et al., the RCAS/t-va system was used to overexpress platelet-derived growth factor B (PDGF-B) in Nestin-expressing progenitors within the neonatal brainstem to promote cell proliferation, combined with Ink4a-ARF deletion which removes tumor suppressor functions, driving glioma formation [45]. This approach successfully generated high-grade brainstem gliomas that recapitulated the genetic and histological features of human tumors [45,187]. In another instance, the overexpression of PDGFB in Nestin-expressing cells within the neonatal brainstem, combined with Ink4a-ARF deletion, resulted in the formation of brainstem gliomas [188].

### 4.3. In Utero Electroporation

In utero electroporation (IUE) has emerged as a powerful tool for delivering oncogenic plasmids directly into a mouse embryo’s ventricular space (lateral or fourth ventricle) [6,37,49,50,71,72,130,189,190,191,192,193]. This method yields fully penetrant cortical and brainstem gliomas in immunocompetent mice, which exhibit a range of histological and molecular features, closely mimicking the progression and pathology observed in human brain tumors. Using IUE models, we have generated tumors in the forebrain and brainstem using the following combination of oncogenes using the PiggyBac transposon system: (i) PDGFRA D842V mutation; (ii) dominant-negative TP-p53; and (iii) co-occurring histone wild-type H3-WT (PPW)- or H3K27M (PPK)/H3G34RV(PPG)-mutant histone genes H3.3 (H3.3A) or H3.1 (H3C2). IUE tumors recapitulate the hallmark features of human DMG and DHG harboring these respective mutations [37,72,93]. Notably, we have established syngeneic cell lines (H3.3/H3.1PPK, PPG, PPW) derived from these tumors, which reliably form gliomas in the brainstem and forebrain following orthotopic re-implantation in immunocompetent C57BL/6 mice [37,50,72]. Although the IUE mouse model involves a technically complex and sensitive surgical procedure, its high value in faithfully recapitulating pediatric brain tumors makes it an important tool for pHGG research [50,72]. To improve accessibility, key optimizations include standardizing plasmid constructs and electroporation protocols to enhance reproducibility and tumor penetrance, incorporating immune-competent mouse strains to better model tumor–immune interactions, and optimizing tumor latency and tracking with reporter genes. Enhancing scalability through high-throughput IUE techniques and automated embryo handling would enable larger, more uniform cohorts. Finally, broader sharing of validated constructs, protocols, and comprehensive tumor characterization data will be essential for widespread adoption and cross-laboratory consistency.

### 4.4. Transposon-Mediated Delivery

Transposon-based delivery systems, such as the SB transposon, offer a robust platform for precise gene integration and expression in glioma models [194,195,196,197]. This approach is particularly advantageous for inducing targeted and regulated gene expression, making it widely applied in the study of pHGGs [198,199]. It is essential, however, to account for off-target effects, which can occasionally occur and complicate the interpretation of the results. Transposon-mediated delivery has successfully facilitated the modeling of pediatric gliomas, particularly through the integration of plasmid DNA into neonatal mouse brain cells. These systems play a significant role in identifying novel genetic drivers associated with tumor development and investigating the functional effects of specific genetic alterations on tumor phenotypes [196,197,198,200,201,202,203,204].

Each preclinical model for pediatric brain tumors possesses distinct strengths and limitations. While GEMMs and viral-based delivery systems have contributed substantially to our understanding of tumor biology, their translation into clinical applications remains limited. Continuous refinement of these models is crucial to achieving breakthroughs in therapeutic strategies for pediatric patients with brain tumors.

### 4.5. Zebrafish Models

Zebrafish (Danio rerio) have emerged as a valuable model organism in pediatric brain tumor research due to their genetic similarity to humans, transparent embryos, and rapid development. These features make zebrafish particularly useful for studying tumor initiation, progression, metastasis, and drug screening in a living organism. Zebrafish tumor models can be created using two primary strategies (Figure 1): xenograft models and syngeneic models.

#### 4.5.1. Xenograft Models

Human patient-derived glioma cells are injected into zebrafish embryos at the one-cell stage or later developmental stages. The transparent nature of zebrafish embryos allows for real-time imaging of tumor cell proliferation, invasion, and angiogenesis. This approach is highly effective for rapid, high-throughput drug screening [140,141,142,143,144].

#### 4.5.2. Syngeneic Models

Syngeneic models use zebrafish-derived brain tumor cells or genetically engineered zebrafish to express key oncogenes or mutations like H3K27M, TP53, and PDGFRA. Techniques such as ZFN (zinc finger nuclease), TALEN (transcription activator-like effector nuclease), or CRISPR-Cas9 can be used to introduce these mutations into zebrafish embryos at the one-cell stage. Following tumor development, brain tumor cells can be re-implanted into adult zebrafish (e.g., Casper strain, which is transparent in adulthood), enabling longitudinal studies of tumor behavior and treatment response [140,141,142,143,144].

Zebrafish models have been instrumental in studying pediatric gliomas like DMGs and evaluating BBB-permeable drugs, providing a cost-effective platform for preclinical testing. The ability to monitor tumor and vascular interactions in real time has significantly advanced the understanding of tumor biology and therapy response. Despite their numerous advantages, zebrafish models present limitations in recapitulating the complex anatomy and adaptive immune system of the human brain. However, their utility in studying TME interactions, genetic pathways, and drug screening makes them an essential tool in pediatric glioma research [140,141,142,143,144].

### 4.6. Syngeneic Allograft Mouse Models

Syngeneic allograft models involve implanting tumor cells derived from an inbred strain into genetically identical hosts, ensuring an immunocompetent environment for evaluating potential therapies. These models are particularly valuable for studying pediatric brain tumors, including DMGs, as they enable the investigation of tumor behavior and immune responses under physiologically relevant conditions [71,72,130]. To advance preclinical immunotherapy research, it is essential to establish mouse models that accurately mimic the genetic, anatomical, and histological traits of human DMG. One widely adopted approach is to generate tumor cell cultures from primary DMG models (e.g., C57BL/6 mice) using IUE [72,130]. These cells are then orthotopically implanted into syngeneic mice to establish allograft models of DMG. Distinct genetically engineered allograft syngeneic models have been developed, including variants expressing H3WT-, H3.3K27M-, and H3.1K27M-mutant subtypes, as well as H3.3G34R/V/-mutant DMGs/DHGs [37,72,190]. These models effectively replicate the histopathological characteristics seen in human DMGs, including diffuse invasive growth patterns and tumor-associated antigen expression.

Furthermore, the immune microenvironment in these mouse models closely resembles that of human DMGs, which typically exhibits significant myeloid cell infiltration and limited presence of T cells and NK cells [205,206]. Studies have demonstrated that murine DMG cells respond to therapies such as HDAC inhibitors, similar to responses observed in patient-derived DMG cells [40,130,207]. These findings underscore the relevance of syngeneic allograft models for preclinical studies, as they accurately simulate the tumor histology, immune landscape, and treatment responses seen in human DMGs.

Despite their advantages, one challenge in immunological research is the lack of comprehensive mouse models that maintain a fully functional immune system while also capturing the molecular and genetic complexity of DMG. Traditional models, such as PDOX in immunodeficient mice, are useful for tumor growth studies but lack immune system functionality, limiting their application in immunotherapy research. Conversely, immunocompetent models, such as carcinogen-induced models or GEMMs, present challenges like extensive breeding and incomplete molecular alignment with human pHGGs biology.

To address these challenges, tumor cell lines have been derived from GEMMs and expanded under in vitro conditions (Figure 2). For example, the SB-transposon system has been instrumental in studying IDH1-mutant gliomas [200]. This method involves knocking down TP53 and ATRX genes while expressing NRASG12V in combination with either wild-type IDH1 or the mutant IDH1-R132H allele [197,198,199,200,202,208]. Furthermore, IUE has been used to introduce PiggyBac vectors, facilitating tumor development. Tumors generated through this method can be cultured ex vivo and subsequently implanted orthotopically to generate syngeneic mouse models. Recent progress has enabled the development of 16 orthotopically engraftable cell lines from IUE-based pHGG models [72]. These cell lines display subtype-specific responses to therapies both in vitro and in syngeneic mouse models. For example, PDGFRA-targeted therapies, such as avapritinib, have shown efficacy in models with PDGFRA amplification (NCT04773782), while hemispheric gliomas harboring the G34R mutation, along with the PDGFRA C235Y variant, respond to infigratinib [72,209,210]. Overall, syngeneic allograft mouse models play a pivotal role in advancing our understanding of pediatric brain tumors. These models provide a robust immunocompetent platform for testing the impact of the TME on immunotherapies and studying tumor biology. By bridging the gap between preclinical research and clinical applications, they offer valuable insights into the strengths and limitations of immunotherapeutic strategies across various pHGGs.

### 4.7. Humanized Mouse Models for Pediatric Brain Tumors

One major limitation of using traditional immunocompromised mouse models for pediatric brain tumor research is the lack of interaction between the tumor and the immune microenvironment. This is critical for understanding tumor biology and developing immunotherapeutic strategies [4,128]. PDX models, while replicating the histological and genetic characteristics of donor tumors, cannot model the tumor–immune microenvironment (TIME) due to the absence of functional immune components [169,175]. To address this issue, humanized xenograft models have been developed [169]. These models involve the co-engraftment of human immune components, such as peripheral blood mononuclear cells (PBMCs), hematopoietic stem cells (HSCs), or activated T cells, along with tumor tissue into highly immunodeficient mouse strains like NSG or NRG mice, which lack innate (NK cells) and adaptive immune cell (B and T cells) activity [169,211,212,213,214]. These models provide valuable platforms for studying human immune responses to tumors and testing immunotherapies (Table 2). However, with few exceptions, no humanized xenograft models have yet been reported specifically for pediatric brain tumors, highlighting an unmet need in this field.

Despite their potential, humanized mouse models present challenges such as cost, complexity, and the risk of complications like graft-versus-host disease (GVHD) [215]. Moreover, they may not fully replicate the unique immune environment of the CNS, which plays a significant role in tumor progression and therapy response. Immunocompetent mouse models could preserve these immune interactions but have been historically limited by species-dependent host-versus-graft rejection. However, recent advances in inducing immune tolerance have enabled the use of immunocompetent mice for human brain tumor research. By selectively inhibiting T-cell activation pathways, such as the interaction of CD80/CD86 with CD28 and CD40 with CD154, researchers have prevented graft rejection and established long-term tumor growth in immunocompetent models [216,217]. This approach has been successfully demonstrated in glioblastoma multiforme (GBM) and DMG models, where selective T-cell co-stimulation blockade allowed for the orthotopic transplantation of human-derived tumors [217].

These models recapitulated the histopathological features of the original tumors and supported long-term tumor growth until disease progression. Such models bridge the gap between PDX and traditional immunocompetent systems and provide an invaluable tool for studying the interactions between tumors and the human immune system in a physiologically relevant context. These advancements offer exciting opportunities for developing and evaluating novel immunotherapies for pediatric brain tumors.

## 5. Large Animal Models for Pediatric Brain Tumor Research

Developing models in larger species, such as pigs or non-human primates, can provide a more accurate representation of human tumor biology and therapeutic responses. These models are particularly useful for studying DMGs and other complex brain tumors (Table 2) [132,135]. Large animal models offer significant advantages for studying pediatric brain tumors due to their anatomical and physiological similarities to humans. These models bridge the gap between rodent studies and human clinical trials, providing a more accurate platform for testing treatments such as surgical resection and adjuvant therapies. Porcine models, in particular, have shown promise due to their gyrencephalic brains, which better mimic the human cortex [218,219]. These models facilitate high-resolution imaging and allow for the study of tumor infiltration and drug delivery within cortical structures. Additionally, spontaneous GBM formation in canines provides a unique opportunity to study glioma in a natural setting, although the rarity and lack of reproducibility of these occurrences pose challenges [220,221,222,223]. Non-human primates offer the closest physiological and genetic similarities to humans, but their use is limited by ethical considerations [224,225]. Overall, porcine models appear to be the most developed and promising for preclinical studies [226], offering a robust platform for investigating new treatments and improving translational outcomes in pediatric brain tumor research.

## 6. In Vitro Models of Pediatric Brain Tumors

### 6.1. 2D and 3D Cultures

In vitro models, including 2D and 3D cultures, provide indispensable platforms for investigating tumor-specific genetic alterations, drug responses, and functional studies [227,228]. Patient-derived cell lines, along with their CRISPR-Cas-corrected isogenic counterparts, enable precise genetic manipulation and high-throughput screening in a controlled environment [56,60,96,127,136,160]. However, while 2D cultures offer convenience and scalability, they often fail to replicate the complex TME and cellular interactions present in vivo (Figure 2).

Over 120 cell lines have been established from various pediatric brain tumors, including pHGGs, DMGs, ependymomas, medulloblastomas, and atypical teratoid rhabdoid tumors (ATRTs) [72,166,229]. A comprehensive overview of pHGG cell lines and their associated histone and co-mutation profiles, as detailed in Table 1 of Furst et al. provides invaluable insights for preclinical modeling and therapeutic studies in these aggressive tumor types [70]. Traditional adherent cultures in serum-containing media can diverge from the original tumor’s genomic and phenotypic features. By contrast, 3D cultures in serum-free conditions, such as neurospheres, better preserve tumor heterogeneity [52,227,230]. Neurospheres maintain a steady state of GSCs alongside differentiated tumor cells, making them a robust model for studying proliferation, differentiation, and therapeutic resistance [69].

Tumoroids, derived directly from fresh tumor samples, transiently retain the heterogeneity of the tumor and its microenvironment [230]. Unlike neurospheres, these models include both malignant and non-malignant cell types, providing an in vitro system closely resembling the TME. However, they can also be used for longer-term studies to investigate tumor progression and resistance [231]. Glioma spheroids, often cultured in suspension, have demonstrated utility in short-term studies of stromal and immune interactions (Figure 2) [69,138,227,230,232].

### 6.2. Brain Organoids

Brain organoids, derived from stem cells, replicate the structural and functional characteristics of the human brain in 3D. These models are pivotal for studying tumor–brain interactions, the TME, and mechanisms of metastatic/invasive behavior under conditions that better mimic human physiology [56,57,58,59,60,61,212,230,232,233,234]. Brain organoids surpass traditional 2D models by offering a 3D architecture that better replicates the in vivo environment, maintaining genetic and molecular heterogeneity essential for brain tumor studies [59,62].

Induced pluripotent stem cell (iPSC)-derived cerebral organoids can self-organize into complex 3D structures, generating both neuronal and non-neuronal cell types [126,232,235,236]. These platforms allow deeper insights into glioma invasiveness and resistance mechanisms. By introducing tumor cells into organoids, researchers can study tumor progression, cellular interactions, and drug response in an accurate and feasible setting (Figure 2). For instance, human cerebral organoids have been used to observe GBM development and its interaction with normal brain cells, paving the way for personalized therapeutic strategies.

### 6.3. Types of Brain Organoids

#### 6.3.1. Glioblastoma Organoids (GBOs)

Generated directly from patient-derived glioblastoma tissues, GBOs retain the genetic and molecular heterogeneity of the original tumors. To develop GBOs, freshly resected tumor tissues are dissociated into single cells and cultured in a medium enriched with growth factors such as EGF and bFGF. These growth factors provide a supportive environment for tumor cell proliferation and heterogeneity. GBOs are invaluable for studying GBM progression and testing personalized therapeutic regimens in a physiologically relevant system [4,57,58,60].

#### 6.3.2. Neoplastic Cerebral Organoids (neoCORs)

Engineered by introducing oncogenic mutations (e.g., RAS or TP53 mutations) into cerebral organoids using CRISPR/Cas9 or lentiviral systems, NeoCORs are cultured in a neurobasal medium supplemented with N2, B27, SHH, and BMP inhibitors. This system models early-stage tumorigenesis and the transition from normal to neoplastic cells. NeoCORs, in contrast to GBOs, are designed to study the initiation of tumorigenesis rather than the dynamics of an established tumor [56].

#### 6.3.3. Glioblastoma-like Cerebral Organoids (GLICOs)

Created by co-culturing GBM cells with cerebral organoids, GLICOs involve embedding organoids in Matrigel and co-culturing with glioblastoma stem cells in a medium containing EGF, bFGF, and heparin. They allow researchers to study tumor invasion, cellular interactions, and glioma-specific mechanisms in a brain-like environment [61,237]. GLICOs are specifically designed to investigate the dynamic interactions between tumor cells and host brain environments, setting them apart from GBOs and neoCORs by focusing on invasion and tumor–host crosstalk.

#### 6.3.4. Tumor-Bearing Organoids (TBOs)

These models are formed by integrating tumor spheroids or patient-derived glioma stem cells into cerebral organoids. TBOs are grown in a Matrigel-based medium with N2, B27, EGF, and bFGF to examine tumor–brain microenvironment interactions, including invasion and resistance mechanisms [60,238]. The Matrigel supports extracellular matrix (ECM) interactions critical for invasion studies.

#### 6.3.5. Patient-Derived Organoids (PDOs)

Directly derived from resected tumor tissues, PDOs closely mimic patient-specific tumor biology. PDOs are developed by culturing dissociated tumor tissues in an organoid medium enriched with EGF, bFGF, and ROCK inhibitor Y-27632. Notably, PDOs have demonstrated sustained expandability while preserving genomic and transcriptomic stability, as well as cellular heterogeneity [239]. PDOs are instrumental in high-throughput drug screening and individualized therapies [57,240]. PDOs stand out due to their direct derivation from patient samples, preserving patient-specific epigenetic and genetic profiles.

#### 6.3.6. Microglia-Containing Brain Organoids (MiCBOs)

This novel model incorporates microglia into cerebral organoids to create a neuroimmune-competent environment. MiCBOs are developed by co-culturing neural progenitor cells with GFP-positive myeloid precursor cells in media supplemented with IL-34 and GM-CSF. The MiCBO–tumor fusion model provides a neuroimmune-competent platform that accurately recapitulates DMG’s infiltration dynamics and microglial interactions within a human brain-like environment. By enabling real-time visualization of tumor–immune interactions, this model offers valuable insights into the role of microglia in DMG progression and serves as a robust system for testing targeted therapies. Its ability to bridge the gap between traditional in vitro models and the clinical setting makes it a promising tool for advancing precision medicine approaches in DMG treatment [241]. Immune-competent brain organoids incorporating functional microglia represent a significant advancement for modeling neuroinflammation and tumor–immune interactions. The recent development of the MiCBO-TF model, a fusion of human microglia-containing brain organoids with H3K27M-mutant DMG spheroids, enables the dynamic study of tumor infiltration and microglia behavior, offering a powerful preclinical platform to investigate therapeutic responses in a physiologically relevant tumor–immune microenvironment [241,242].

#### 6.3.7. Medulloblastoma Organoids

These organoids are used in lineage tracing, co-culture, and in vivo models to study the origins and therapeutic responses of pediatric tumors like medulloblastoma. They require media containing SHH, BMP inhibitors, and tailored growth factors [243,244,245]. These models provide insights into specific oncogenic drivers, such as MYC overexpression, and therapeutic vulnerabilities.

#### 6.3.8. Expanded Neuroepithelium Organoids (ENOs)

ENOs are created using temporal morphogen gradients, improving cortical specification. TGF-β and BMP inhibitors enhance neuroepithelial layer formation, making these models superior for cortical development and early-stage studies [246].

Recent advancements include location-specific growth media tailored to the cerebrum, cerebellum, and brainstem, improving organoid fidelity [247]. For example, SHH and BMP inhibitors are used for cerebellar organoids, while forebrain organoids benefit from TGFβ inhibitors and Wnt antagonists. These tailored media improve region-specific tumor modeling [234,247]. In parallel, models like S100b^+^ progenitor-driven organoids emphasize the role of specific progenitor populations in cancer initiation [245]. Media enriched with IL-34 and GM-CSF, alongside Notch pathway modulation, are critical for generating such models, enabling the study of early transformation events. Using dorsal forebrain organoids from human iPSCs, researchers identified quiescent GBM cells, tracked via fluorescent reporters, and tested harmine, a DYRK1A/B inhibitor, for its therapeutic effects [248].

Limitations remain with these models, including the absence of complete vascularization, immune cell components, and long-term viability [58,249]. By addressing these challenges, brain organoid models continue to bridge critical gaps in glioblastoma research, enabling the study of tumor biology, therapeutic vulnerabilities, and drug response in a realistic setting (Figure 2). For example, the integration of endothelial cells engineered to express human ETS variant 2 (ETV2) has shown potential in creating vascular-like networks that enhance BBB characteristics and tissue maturation [250].

## 7. Ex Vivo Models

Ex vivo models, such as organotypic brain slice cultures, provide a unique platform to maintain the native tissue architecture, enabling detailed studies of tumor invasion, microenvironmental interactions, and therapeutic responses [251,252,253]. Brain slices derived from rodents or transgenic mice preserve the cytoarchitecture and vascular cells of the tissue, making them valuable for co-culture experiments. Tumor spheroids or glioma stem cells can be introduced into these slices to study infiltration and migration patterns over several weeks [204,251]. Live-cell imaging allows detailed tracking of cell behavior and lineage, offering insights into the dynamic interplay between tumor cells and their environment [196,204,251].

The viability of organotypic cultures is typically limited to three weeks, posing challenges for long-term studies, particularly with slower-growing glioma stem cells [253,254]. Despite the absence of active blood flow, these models maintain their native characteristics, allowing researchers to examine tumor–glia interactions and microenvironmental dynamics with minimal artifacts. Despite their technical demands and low throughput, these models remain invaluable for mechanistic studies of tumor behavior [255]. Their utility for mechanistic studies makes them valuable for an understanding of glioma biology, epigenetic dysregulation, and therapeutic vulnerabilities (Figure 2).

Recent advancements, such as 3D bioprinting and microfluidic devices, have further enhanced the physiological relevance of glioma models: 3D bioprinting integrates glioma stem cells, immune cells, and ECM components to recreate the TME and study therapeutic responses. Microfluidic devices replicate the dynamic TME by simulating BBB functionality and cellular interactions [53,139,228,230]. Together, these models offer unparalleled insights into glioma biology, bridging the gap between experimental findings and clinical translation (Figure 2).

## 8. Advances in Tumor Detection Methods

Several new techniques in tumor imaging have been developed to improve our ability to diagnose CNS tumors and evaluate their response to therapy. Multiparametric magnetic resonance imaging (MRI), utilizing parameters beyond the standard T1- and T2-weighted scans, with diffusion-weighted imaging (DWI), the apparent diffusion coefficient (ADC), and perfusion-weighted imaging to help better distinguish tumor from non-tumor on imaging, is commonly utilized in clinical settings, along with magnetic resonance (MR) spectroscopy in some cases [256]. A few other parameters are also under investigation [257]. Diffusion tensor imaging in MR allows for the characterization of white matter tracts [258], particularly useful for surgical planning to help avoid “eloquent cortex” [259]. Chemical exchange saturation transfer (CEST) is an emerging technique that conveys chemical information, such as the relative proportion of amide groups in tissue, to assist with differentiating tumor from pseudo-progression/radiation necrosis [260], similarly to amino-acid positron emission tomography (PET)–MRI [261,262,263]. Metabolomic data, assessed via hyperpolarized radiolabeled C13-MRI, has been similarly studied with some promising results [264], though, as is true for many of the above-mentioned methods, it has not yet entered common clinical practice. Radiomics, the utilization of radiographic data to ultimately leverage machine learning models in radiology, including the above parameters, is another area under heavy investigation [265,266]. Diagnosis still requires tissue sampling, with advances in biopsy techniques including MR- and computed tomography (CT)-guided stereotactic biopsy allowing for tissue sampling in difficult anatomic locations, such as lesions intrinsic to the brainstem [267]. Liquid biopsy of high-grade gliomas has shown promise in terms of the molecular characterization of tumors via CSF and even plasma sampling, with increases in cell-free tumor DNA tending to occur prior to radiographic progression [268,269,270,271]. Liquid biopsy with CSF sampling is incorporated into the exploratory objectives of several current early-phase pHGG clinical trials, including methods to increase the yield of liquid biopsy via BBB disruption with focused ultrasound [272].

## 9. Future Directions

The establishment of organoid models specific to midline brain structures, such as the pons, represents a critical step in advancing in vitro studies of tumor–brain interactions, particularly for midline gliomas like DMGs. These organoid models can be generated using directed differentiation protocols, incorporating growth factors and morphogens (e.g., SHH, FGF, and BMP signaling pathways) to replicate the specific regional identity of the midline brain [57,244]. By utilizing lipofectamine-mediated transfection and electroporation, genetic lesions including H3K27M, PDGFRA (D842V), DNp53, and EGFRvIII, as well as combinations such as H3K27M-DNp53-PDGFRA, can be introduced into iPSCs. These genetically engineered iPSCs can then be differentiated into 3D organoid models that faithfully recapitulate the genomic and phenotypic characteristics of DMGs. Such models allow for the detailed investigation of tumor initiation, progression, and interaction with the brain microenvironment, including neuronal and glial cell components. Moreover, these models serve as a robust platform for the preclinical testing of targeted therapies and studying the dynamic interplay between tumor cells, stromal components, and immune infiltrates within a physiologically relevant system.

Recent advancements in immune-competent GEMMs provide a robust platform for modeling the human immune–tumor interface. Integrating GEMM-derived tumor cells into 3D neural organoids offers an innovative approach to simulate the TIME. These hybrid systems can incorporate vascularization and immune cell populations, recapitulating features of the in vivo tumor milieu. For example, vascularization strategies, such as co-culture with endothelial cells or genetic induction of vascular-like structures using ETV2, have shown promise in overcoming the hypoxic limitations of traditional organoids [250]. Furthermore, incorporating immune cells derived from GEMMs, including myeloid-derived suppressor cells and tumor-infiltrating lymphocytes, can provide critical insights into the immune-modulatory effects of experimental therapies.

The integration of cutting-edge techniques, such as microelectrode array (MEA) recording, calcium imaging, and transcriptomic analysis, can enhance the functional characterization of tumor organoids. These methodologies allow for real-time monitoring of neural activity, synaptic connectivity, and epigenetic changes induced by therapeutic interventions. The ability to fuse tumor organoids with region-specific brain organoids, such as thalamic or cortical organoids, could further elucidate the mechanisms underlying tumor infiltration and regional specificity in glioma progression.

Combining high-throughput screening with advanced gene-editing technologies, such as CRISPR-Cas9, enables the identification of key genetic drivers and therapeutic targets. Organoid platforms can be utilized to evaluate the efficacy of novel drug combinations targeting mutant H3K27M and associated epigenetic alterations. The integration of isogenic organoid models allows for comparative studies between mutant and corrected phenotypes, providing a powerful tool for identifying context-specific therapeutic vulnerabilities.

To enhance the translational potential of organoid models, future efforts must address limitations such as heterogeneity, limited vascularization, and the lack of long-term culture viability. Advances in bioreactor technologies, the use of slicing methods to improve nutrient and oxygen diffusion, and the incorporation of human-derived vascular structures are pivotal for achieving physiological relevance. Moreover, the co-culture of tumor organoids with patient-derived immune cells or cerebral microvascular cells can offer a more comprehensive understanding of therapy resistance and tumor–immune dynamics.

## 10. Conclusions and Prospects

This review emphasizes the critical need for innovative and physiologically relevant model systems to advance our understanding of pediatric brain tumors. While existing in vitro and ex vivo models, including patient-derived cell lines, organoids, and organotypic brain slices, have provided significant insights into tumor biology, they are often limited in their ability to fully recapitulate the complexities of the TME, including immune interactions and vascularization.

Emerging technologies, such as patient-specific organoids, CRISPR-Cas9 gene editing, and 3D bioprinting, have the potential to address these limitations. Midline brain organoid models and advanced GEMM-derived organoid systems can serve as transformative platforms for studying tumor–brain interactions, immune–tumor dynamics, and therapeutic resistance. These models offer unprecedented opportunities for high-throughput drug screening, personalized treatment development, and understanding the molecular mechanisms driving pediatric brain tumor progression.

Looking forward, the integration of multi-omics approaches (e.g., single-cell RNA-seq, proteomics, and epigenomics) with these advanced models will provide a systems-level understanding of tumor biology. Additionally, incorporating vascularization and tissue-resident immune cells into organoid models will further improve their translational relevance, bridging the gap between preclinical studies and clinical applications.

Ultimately, leveraging these advanced preclinical models is vital to overcoming the current barriers in pediatric neuro-oncology research. By aligning laboratory innovations with clinical needs, we can accelerate the development of effective, targeted therapies, offering hope for improved outcomes and quality of life for children with brain tumors. Altogether, innovative model systems are crucial for translating preclinical findings into effective pediatric brain tumor therapies.

## Figures and Tables

**Table 1:** Overview of pediatric brain tumor subtypes, detailing genetic alterations, age groups, prevalence, prognosis, and 5-year overall survival (OS) rates.

**Pediatric High-Grade Gliomas (pHGGs)**					
**Subtype**	**Genetic Alterations**	**Age Groups**	**Prevalence**	**Prognosis**	**5-Year OS**
**Diffuse midline gliomas (DMGs)**	*TP53; H3.1/3K27M; NF1; ACVR1; PIK3CA; FGFR; PDGFRA*	Younger children (H3.1) (age range: <3–7 years) and teens (H3.3)	40%	Worst	<1%
**Diffuse hemispheric gliomas (DHGs)**	*TP53; H3.3G34R/V; ATRX; PDGFRA*	Older children (age range: 6–15 years) and young adults	10%	Poor	<5%
**Infantile receptor tyrosine kinase (RTK) fusion glioma**	*ALK; ROS1; MET; NTRK fusions*	Infants (<3 years)	15–20%	Intermediate	~42.9–53.8%
**H3-wt/IDH-wt**	*TP53; MYCN; PDGFRA;* *EGFR*	Children (3–10 years) and young adults	50%	Intermediate; poor	≤5%
**Pediatric Low-Grade Gliomas (pLGGs)**					
**IDH-mutant glioma**	*IDH1/IDH2 mutant*	Adolescents and young adults (15–30 years)	<5%	Better	30–40%
**Pilocytic astrocytoma (PA)**	*BRAF-KIAA1549 fusion, NF1 loss*	5–15 years	30–40%	Excellent	>95%
**Diffuse astrocytoma (MYB/MYBL1)**	*MYB/MYBL1 rearrangements*	10–20 years	~5%	Good	~85–95%
**Ganglioglioma**	*BRAF V600E mutation*	5–20 years	~10%	Favorable	~80–95%
**Pleomorphic xanthoastrocytoma**	*BRAF V600E mutation*	10–25 years	1–3%	Favorable	~70–80%
**Subependymal giant cell astrocytoma**	*TSC1/TSC2 mutations*	Infants and young children (0–10 years)	~1–2%	Excellent (mTOR inhibitors)	>95%
**Dysembryoplastic neuroepithelial tumor**	*FGFR1 alterations*	5–20 years	<1–2%	Excellent	>95%
**Pilomyxoid astrocytoma**	*BRAF alterations*	Infants and young children (0–5 years)	~1–2%	Worse than PA	~80%
**Ependymoma**					
**Hemispheric supratentorial-RELA (ST-RELA)**	*ZFTA-RELA; ZFTA-YAP1; ZFTA-MAML2*	Children (3–10 years)	18%	Poor	<70%
**Hemispheric supratentorial-YAP1 (ST-YAP1)**	*YAP1-MAMLD1; YAP1-FAM118B*	Infants (<3 years)	3%	Good	>90%
**Cerebellar posterior fossa-A (PF-A)**	EZHIP; MAP3K20; TGA6; Chr 1q gain or 6q loss	Children (3–10 years)	48%	Poor	70–85%
**Cerebellar posterior fossa-B (PF-B)**	Chromosomal instability; H3K27M	Teens (age range: 13–19 years) and adults	10%	Good	>90%
**Spinal cord (SP-EPN: spinal ependymoma)**	MYCN; NF2; chromosomal instability; Chr 22q loss	Adults	4%	Good	>70%
**Medulloblastoma (MB)**					
**WNT-activated (WNT-MB)**	*TP53; CTNNB1; SMARCA4; DDX3X*	Older children (age range: 6–15 years) and adults	10%	Best	95%
**Sonic hedgehog-activated** **(SHH-MB)**	*TP53; PTCH1; SMO; MYCN; GLI1; GLI2; SUFU; MLL2*	Infants (<3 years), children (3–10 years), and adults	30%	Intermediate; poor	75%
**Group-3 MB**	*MYC/MYCN; OTX2; MLL2; CHD7*	Infants (<3 years) and younger children (age range: <3–7 years)	25%	Very poor	50%
**Group-4 MB**	**Group-3 MB +** *CDK6; KDM6A; UTX; PRDM6; CBFA complex; DDX31; GFI1/GFI1B; KMT2C; MLL3*	Infants (<3 years), older children (age range: 6–15 years), and adults	35%	Intermediate, poor	75%

**Table 2:** Summary of strengths and limitations of preclinical models of pediatric brain tumors, including in vitro, in vivo, and ex vivo systems.

Preclinical Pediatric Brain Tumor Model	Strengths/Advantages	Limitations/Disadvantages
**In vitro 2D cell culture**	Cost-effective, rapid drug screening, enables study of specific molecular mechanisms.	Does not represent tumor heterogeneity, lacks microenvironmental interactions and hypoxic regions.
**Neurosphere cultures**	Maintains tumor heterogeneity, retains tumor genotype, preserves stem-like properties.	Requires enriched medium, limited scalability, stem-like cells grow disproportionately.
**Patient-derived xenografts (PDXs)**	Maintains tumor histological features, versatile for drug screening and toxicity studies.	Engraftment rate is variable, lacks immune system contribution in immunodeficient models.
**Genetically engineered mouse models (GEMMs)**	Replicates tumor initiation in vivo, includes immune interactions, supports blood–brain barrier integrity and tumor microenvironment (TME).	Species differences limit translational relevance, costly, lacks complete tumor heterogeneity.
**In utero electroporation (IUE) GEMMs**	Allows precise genetic manipulation, recapitulates human-like tumor features, syngeneic models for immune-competent studies. IUE–PiggyBac system able to carry significantly larger cargo, typically up to 100 kb, making it highly suitable for transducing larger oncogene constructs or multiple genes simultaneously.	Although offering large cargo capacity, PiggyBac often considered more complex in terms of the delivery process and optimization.
**Sleeping Beauty transposon (SB-GEMMs)**	Stable and controlled gene integration, ideal for studying specific genetic mutations in gliomas.	Risk of off-target effects, labor-intensive validation. SB system typically carrying cargo of up to ~10–15 kb, which is relatively smaller compared with PiggyBac.
**Syngeneic allograft models**	Supports immune-competent studies, replicates tumor histology and immune response.	Limited molecular alignment with human tumors, challenging standardization protocols.
**Humanized mouse models**	Facilitates study of human tumor–immune interactions, suitable for testing preclinical immunotherapies.	Expensive, risk of graft-versus-host disease (GVHD), incomplete central nervous system (CNS)-specific immune response.
**Zebrafish brain tumor models**	Cost-effective and high-throughput screening for drug discovery and testing, transparent embryos allow real-time imaging of tumor growth, invasion, angiogenesis. Rapid tumor development and shorter experimental timelines compared to mammalian models.	Limited physiological and anatomical similarity to the human brain, lack of a mature adaptive immune system in early developmental stages, affecting immunotherapy studies. Smaller brain size restricting the ability to model complex tumor behaviors. Ethical and technical considerations for scaling to advanced therapeutic interventions.
**Ex vivo models (brain slice cultures)**	Retains native tissue architecture, enables study of tumor invasion and cellular interactions.	Short viability, lacks active blood flow, limited to small-scale studies.
**Large animal models**	Physiological similarity to humans, ideal for drug delivery and tumor infiltration studies.	High cost, ethical and logistical challenges.
**CRISPR-Cas9-engineered models**	Precise genetic editing for specific mutations, relevant for studying H3K27M and other targets.	Risk of off-target effects, requires advanced expertise.
**Tumor organoids**	Recapitulates tumor architecture, hypoxic gradients, useful for biomarker testing.	Absence of vasculature, host immune cells, limited cell diversity, technically demanding.
**Cerebral organoids**	Human-like brain microenvironment, retains heterogeneity and tumor invasiveness.	Lacks mature brain tissue, no immune compartment, resembles fetal brain structures.
**Advanced organoids with vascularization**	Incorporates endothelial and immune components, enhances physiological relevance.	Technically demanding, scalability issues, high cost.
**Microfluidic devices**	Mimics TME dynamics, compartmentalization increases reproducibility.	Complex and costly, not standardized, typically uses pre-differentiated cell types.
**3D bioprinting**	Enables precise spatial control, supports co-culture of various cell types, mimics extracellular matrix interactions.	Requires high technical expertise, variability between bioinks, increased cost.

## Data Availability

Not applicable.

## Abbreviations

The following abbreviations are used in this manuscript:

pHGG	pediatric high-grade glioma
DMG	diffuse midline glioma
TME	tumor microenvironment
BBB	blood–brain barrier
NPC	neural precursor cell
OPC	oligodendrocyte precursor cell
GSC	glioma stem cell
BTB	blood–brain tumor barrier
PDX	patient-derived xenograft
GEMM	genetically engineered mouse model
CNS	central nervous system
WHO	World Health Organization
*NF1*	neurofibromatosis type 1
*NF2*	neurofibromatosis type 2
DIPG	diffuse intrinsic pontine glioma
OS	overall survival
pLGG	pediatric low-grade glioma
DHG	diffuse hemispheric glioma
RTK	receptor tyrosine kinase
PA	pilocytic astrocytoma
ST-RELA	supratentorial-RELA
ST-YAP1	supratentorial-YAP1
PF-A	posterior fossa-A
PF-B	posterior fossa-B
SP-EPN	spinal ependymoma
MB	medulloblastoma
NGS	next-generation sequencing
IUE	in utero electroporation
SB	Sleeping Beauty
ZFN	zinc finger nuclease
TALEN	transcription activator-like effector nuclease
PXA	pleomorphic xanthoastrocytoma
iPSC	induced pluripotent stem cell
EB	embryoid body
ECM	extracellular matrix
NSC	neural stem cell
RSV-1	Rous sarcoma virus-1
Ad12	human adenovirus 12
CDX	cell line-derived xenograft
PDOX	patient-derived orthotopic xenograft
GVHD	graft-versus-host disease
BTIC	brain tumor-initiating cell
PDX-MI	PDX Minimal Information standard
PDGF	platelet-derived growth factor
TIME	tumor–immune microenvironment
PBMC	peripheral blood mononuclear cell
HSC	hematopoietic stem cell
GBM	glioblastoma multiforme
ATRT	atypical teratoid rhabdoid tumor
GBO	glioblastoma organoid
neoCOR	neoplastic cerebral organoid
GLICO	glioblastoma-like cerebral organoid
TBO	tumor-bearing organoid
PDO	patient-derived organoid
MiCBO	microglia-containing brain organoid
ETV2	ETS variant 2
ENO	expanded neuroepithelium organoid
MEA	microelectrode array
MRI	magnetic resonance imaging
DWI	diffusion-weighted imaging
ADC	apparent diffusion coefficient
MR	magnetic resonance
CEST	chemical exchange saturation transfer
PET	positron emission tomography
CT	computed tomography
